# 30.2 GENETIC VARIATION RELATED TO IMMUNE FUNCTION AND SCHIZOPHRENIA RISK: EVIDENCE FOR EFFECTS ON COGNITION

**DOI:** 10.1093/schbul/sby014.123

**Published:** 2018-04-01

**Authors:** Gary Donohoe

**Affiliations:** NUI Galway

## Abstract

**Background:**

Altered immune response is associated with many psychiatric disorders, but whether and how these changes confer increased risk remains unclear. In schizophrenia, robust association between illness risk and the MHC region general, and complement component 4 (C4) specifically, has been demonstrated, along with evidence from both gene enrichment and other genetic analysis highlighting the broader role of genetic variation in additional immune related networks to schizophrenia risk.

**Methods:**

In a series of recent studies from our group, we examined the effects of immune-related genetic variation, based on gene ontology, implicated in neural function both behaviourally in samples of ~1200 cases and controls, and cortically in samples of ~150 cases and controls.

**Results:**

We found that (1) increased predicted C4A RNA expression predicted poorer performance on measures of memory recall (p=0.016, corrected) and a pattern of reduced cortical activity in middle temporal cortex during a measure of visual processing (p<0.05, corrected); (2) variation in a curated gene set associated with both increased Schizophrenia risk and immune function (CSMD1, DPP4, SRPK2, TRIM8, STAT6, FES, EP300, TNFRSF13c) were associated with both variation in both episodic memory and general cognitive ability.

**Discussion:**

Based on these findings we conclude that schizophrenia risk associated with variation within immune related genes is likely to be conferred at least partly via effects on cognition, and the molecular mechanisms involved may include effects on inflammatory response.

